# Fetal cardiac dysfunction in intrahepatic cholestasis of pregnancy is associated with elevated serum bile acid concentrations

**DOI:** 10.1016/j.jhep.2020.11.038

**Published:** 2021-05

**Authors:** Tharni Vasavan, Sahil Deepak, Indu Asanka Jayawardane, Maristella Lucchini, Catherine Martin, Victoria Geenes, Joel Yang, Anita Lövgren-Sandblom, Paul Townsend Seed, Jenny Chambers, Sophia Stone, Lesia Kurlak, Peter Hendy Dixon, Hanns-Ulrich Marschall, Julia Gorelik, Lucy Chappell, Pam Loughna, Jim Thornton, Fiona Broughton Pipkin, Barrie Hayes-Gill, William Paul Fifer, Catherine Williamson

**Affiliations:** 1 Department of Women and Children’s Health, King's College London, London, UK; 2 University Department of Obstetrics and Gynaecology, Nottingham City Hospital, University of Nottingham, Nottingham, UK; 3 Faculty of Engineering, University of Nottingham, Nottingham, UK; 4 Departments of Psychiatry and Pediatrics, Columbia University, New York, USA; 5 Department of Laboratory Medicine, Karolinska Institute, Stockholm, Sweden; 6 Women’s Health Research Centre, Imperial College London, London, UK; 7 Department of Obstetrics and Gynaecology, Western Sussex Hospitals NHS Foundation Trust, West Sussex, UK; 8 Department of Molecular and Clinical Medicine, Sahlgrenska Academy, University of Gothenburg, Sweden; 9 Imperial College London, National Heart and Lung Institute, Imperial Centre for Experimental and Translational Medicine, London, UK

**Keywords:** Pregnancy, Female, Ursodeoxycholic acid, Intrahepatic cholestasis of pregnancy, Cholic acid, Stillbirth, Heart rate, Bile, Ventricular dysfunction

## Abstract

**Background & Aims:**

Intrahepatic cholestasis of pregnancy (ICP) is associated with an increased risk of stillbirth. This study aimed to assess the relationship between bile acid concentrations and fetal cardiac dysfunction in patients with ICP who were or were not treated with ursodeoxycholic acid (UDCA).

**Methods:**

Bile acid profiles and NT-proBNP, a marker of ventricular dysfunction, were assayed in umbilical venous serum from 15 controls and 76 ICP cases (36 untreated, 40 UDCA-treated). Fetal electrocardiogram traces were obtained from 43 controls and 48 ICP cases (26 untreated, 22 UDCA-treated). PR interval length and heart rate variability (HRV) parameters were measured in 2 behavioral states (quiet and active sleep).

**Results:**

In untreated ICP, fetal total serum bile acid (TSBA) concentrations (r = 0.49, *p =* 0.019), hydrophobicity index (r = 0.20, *p =* 0.039), glycocholate concentrations (r = 0.56, *p =* 0.007) and taurocholate concentrations (r = 0.44, *p =* 0.039) positively correlated with fetal NT-proBNP. Maternal TSBA (r = 0.40, *p =* 0.026) and alanine aminotransferase (r = 0.40, *p =* 0.046) also positively correlated with fetal NT-proBNP. There were no significant correlations between maternal or fetal serum bile acid concentrations and fetal HRV parameters or NT-proBNP concentrations in the UDCA-treated cohort. Fetal PR interval length positively correlated with maternal TSBA in untreated (r = 0.46, *p =* 0.027) and UDCA-treated ICP (r = 0.54, *p =* 0.026). Measures of HRV in active sleep and quiet sleep were significantly higher in untreated ICP cases than controls. HRV values in UDCA-treated cases did not differ from controls.

**Conclusions:**

Elevated fetal and maternal serum bile acid concentrations in untreated ICP are associated with an abnormal fetal cardiac phenotype characterized by increased NT-proBNP concentration, PR interval length and HRV. UDCA treatment partially attenuates this phenotype.

**Lay summary:**

The risk of stillbirth in intrahepatic cholestasis of pregnancy (ICP) is linked to the level of bile acids in the mother which are thought to disrupt the baby’s heart rhythm. We found that babies of women with untreated ICP have abnormally functioning hearts compared to those without ICP, and the degree of abnormality is closely linked to the level of harmful bile acids in the mother and baby’s blood. Babies of women with ICP who received treatment with the drug UDCA do not have the same level of abnormality in their hearts, suggesting that UDCA could be a beneficial treatment in some ICP cases, although further clinical trials are needed to confirm this.

## Highlights

• ICP is associated with an abnormal fetal cardiac phenotype.
• Phenotype includes increased PR interval, heart rate variability and NT-proBNP level.
• Degree of abnormality is associated with fetal and maternal total serum bile acid levels.
• NT-proBNP level in particular is associated with fetal bile acid hydrophobicity index.
• UDCA treatment partially ameliorates this phenotype.

## Graphical abstract

**Figure:**
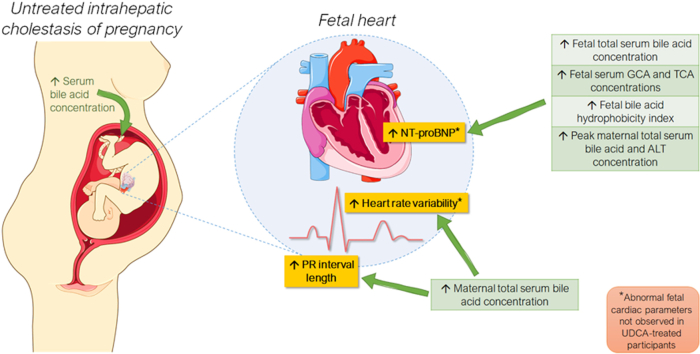

## Introduction

Intrahepatic cholestasis of pregnancy (ICP), the most common gestational liver disease, is diagnosed in women with pruritus and elevated maternal total serum bile acid (TSBA) concentrations.^1^^,^^2^ ICP is associated with adverse pregnancy outcomes; when maternal TSBA concentrations were ≥40 μmol/L the likelihood of spontaneous preterm birth, prolonged neonatal unit admission and fetal asphyxia were significantly increased in a prospective Swedish cohort, and stillbirth was also increased in a UK cohort.^2^^,^^3^ More recently, the prevalence of stillbirth was shown to increase from 0.28% to 3.44% in singleton pregnancies with maternal TSBA concentrations of ≥100 μmol/L; the prevalence of stillbirth in the control cohort of this study was found to be 0.31%.^4^

The mechanism of ICP-associated stillbirth is unknown, with post-mortem findings indicating that infants are appropriately grown.^5^ However, there is evidence of fetal cardiac dysfunction in pregnancies complicated by ICP, with speculation of a sudden arrhythmic event causing fetal demise.^6^ Echocardiography has demonstrated fetal atrial flutter and supraventricular tachycardia in ICP and cardiotocography (CTG) monitoring has detected bradycardia preceding a stillbirth.[7], [8], [9], [10] In women with both ursodeoxycholic acid (UDCA)-treated and untreated ICP, fetal left ventricular (LV) function is impaired, as evidenced by increased LV myocardial performance index, increased myocardial tissue velocities, reduced LV global strain rate and increased isovolumetric contraction and relaxation times via echocardiography, all of which are more pronounced in severe ICP.[11], [12], [13], [14], [15] In addition, concentrations of N-terminal pro-B-type natriuretic peptide (NT-proBNP) and cardiac troponin I, markers used to diagnose heart failure and LV systolic dysfunction, are increased in fetal umbilical venous blood from ICP pregnancies.^15^^,^^16^ There is echocardiography evidence of increased fetal PR interval length in both UDCA-treated and untreated ICP which is associated with disease severity.[17], [18], [19] Although there is currently no evidence to suggest ICP prolongs fetal QTc interval length, maternal QTc interval prolongation has been reported in women with mild ICP.^20^ There are no conclusive data about the effect of ICP on fetal heart rate variability (fHRV), an established indicator of fetal autonomic nervous system function and wellbeing *in utero*, or the effect of UDCA treatment on this parameter.^21^ Fetal heart rate activity exists in 4 different behavioral states (1F-4F), defined based on specific heart rate patterns, eye and body movements.^22^ The fetus spends the majority of the time in 1F and 2F, thought to resemble non-REM (quiet) sleep and REM (active sleep), respectively.^23^ Analyzing fHRV in the context of behavioral state is necessary in order to differentiate between normal and abnormal activity. For example, state 1F is associated with reduced HRV and movement compared to state 2F.^22^

UDCA marginally reduces maternal pruritus and most studies have shown improved concentrations of maternal and fetal TSBA in ICP.^24^^,^^25^ UDCA has a demonstrably lower bile acid hydrophobicity index (HI), a measure associated with bile acid-induced intra- and extracellular cytotoxicity.^26^^,^^27^ It has a protective effect in *in vitro* fetal heart models, attenuating taurocholic acid (TCA)-induced slowing of ventricular conduction velocity in neonatal rat hearts and inhibiting TCA-induced conduction abnormalities in human fetal and adult heart models.[28], [29], [30] The impact of UDCA on human fetal cardiac dysfunction *in vivo*, however, is yet to be fully established. There have been reports of fetal demise and CTG abnormalities occurring in UDCA-treated pregnancies and fetuses of UDCA-treated cohorts have displayed LV dysfunction and prolonged mechanical PR interval length,^10^^,^^12^^,^^17^^,^[30], [31], [32] however most studies do not adjust for severity of hypercholanaemia.

We aimed to establish whether untreated severe ICP causes a fetal cardiac phenotype that predisposes to potentially fatal cardiac arrhythmia using measurement of fetal cardiac time interval (CTI), fHRV and NT pro-BNP concentrations in the umbilical venous blood. We also aimed to evaluate the impact of UDCA treatment on fetal cardiac parameters.

## Patients and methods

### Recruitment for umbilical venous blood assays

**Fig. 1:**
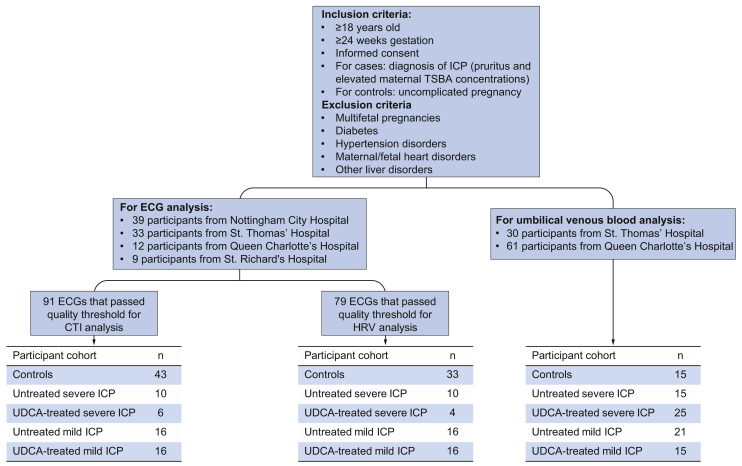
Flowchart depicting numbers of participants in each analyzed cohort and the sites they were recruited from.

Umbilical venous blood was collected from ICP participants ± UDCA treatment and matched controls after informed consent from St. Thomas’ and Queen Charlotte’s Hospitals, UK, between September 2009 and January 2019 (ethical approval REC numbers 15/WM/0017 and 08/H0707/21) (Fig. 1). Some participants were also recruited from the PITCHES trial (n = 14) (EudraCT number: 2014-004478-41).^25^

ICP was diagnosed in women with pruritus and non-fasting serum TSBA concentrations ≥10 μmol/L. Peak maternal TSBA concentrations taken during pregnancy were used to classify women as having mild (10–39 μmol/L) or severe (≥40 μmol/L) ICP. Women treated with UDCA (500–2,500 mg per day) were analyzed separately to untreated cases. Women recruited as controls had uncomplicated pregnancies with no symptoms of ICP and no diagnosis of ICP in previous pregnancies. Maternal and fetal demographic and delivery details for these participants are summarized in Table S1.

Umbilical venous blood was collected immediately after delivery in plain vacutainers and centrifuged for 15 minutes at 3,500 rpm; umbilical serum was frozen within 30 minutes at -80°C. NT-proBNP was assayed using a human proBNP ELISA kit (Raybiotech, GA, USA) as per manufacturer’s instructions. Individual bile acids were measured by ultra-performance liquid chromatography tandem mass spectrometry (UPLC-MS/MS). The HI of fetal bile acids in each serum sample was calculated using the molar fraction and the previously reported hydrophobicity of individual bile acids by Heuman *et al.*.^26^

### Recruitment for ECG recording

Prospective cohorts of women with ICP or uncomplicated pregnancy who were ≥20 weeks of gestation were recruited after informed consent from Nottingham City Hospital between October 2007 and January 2011 and St. Thomas’, Queen Charlotte’s and St. Richard’s Hospitals in the UK between January 2015 and June 2019 (REC numbers 08/H0707/21 and 15/WM/0017) (Fig. 1). Some participants were also recruited from the PITCHES trial (n = 3).^26^ Although ethical permission was granted to collect umbilical venous blood in women who underwent electrocardiogram (ECG) recording, a separate cohort was required due to the low number of women who gave both types of sample. ICP was diagnosed as previously described. Maternal TSBA concentrations were tested via enzymatic assay within 3 days of participation to obtain a measurement close to the time of fetal ECG (fECG) recording. ICP cases treated with UDCA (500–2,500 mg per day) at the time of fECG acquisition were analyzed separately to untreated cases. Demographic and delivery details of participants are shown in Table S2.

### ECG collection, data processing and analysis

The Monica AN24 (Monica Healthcare Limited, Nottingham, UK) was used to obtain overnight fECG recordings as previously described.^33^^,^^34^ ECG processing and analysis are described in the supplementary methods.

### Statistical analyses

Statistical analysis and figures were created using Stata IC v15.0 (Stata Corporation, TX, USA) and are described in the supplementary methods. Initial investigation of differences between laboratory, clinical and delivery details of women who had ECGs or bile acid profile and NT-proBNP measurements was performed via a Kruskal Wallis analysis of variance (ANOVA) without pairwise multiple comparison tests after participants were designated into control, untreated and UDCA-treated cohorts of mild or severe ICP. *p* values of <0.05 were considered statistically significant and data are presented as median [IQR] or n (%).

Partial correlation analysis was used to investigate the strength of association between fetal NT-proBNP concentration and fetal bile acid profiles or maternal TSBA, alanine aminotransferase (ALT) and bilirubin concentrations in untreated and UDCA-treated ICP. Partial correlation analysis was also used to investigate the strength of associations among cardiac time intervals, HRV parameters and maternal TSBA concentration at the time of ECG recording in untreated and UDCA-treated ICP. Skewness and kurtosis tests confirmed all continuous variables were abnormally distributed and were therefore log-transformed (using natural logarithm) prior to analysis. Correlations investigating fetal NT-proBNP concentrations were controlled for the covariates of mode of delivery, induction of labor, gestational age at delivery and fetal sex. Correlations investigating fetal cardiac data were controlled for the covariates of gestational age and fetal sex. Correlations investigating maternal cardiac data were controlled for the covariates of maternal BMI and maternal age.

Fetal HRV data were separated by behavioral state; the small sample size and lower likelihood of finding the fetus in behavioral states 3F or 4F prevented the statistical analysis of these data and they were therefore excluded.

## Results

### Clinical and demographic details of participants

Clinical and demographic details of participants are provided in Tables S1 and S2. Participants with ICP had increased rates of induced labor in both groups compared to controls with uncomplicated pregnancy, and infants from participants with severe ICP, in whom fECG was collected, had increased neonatal unit admission.

### Maternal and fetal serum bile acid concentrations and fetal bile acid HI are positively correlated with fetal NT-proBNP concentrations

**Fig. 2:**
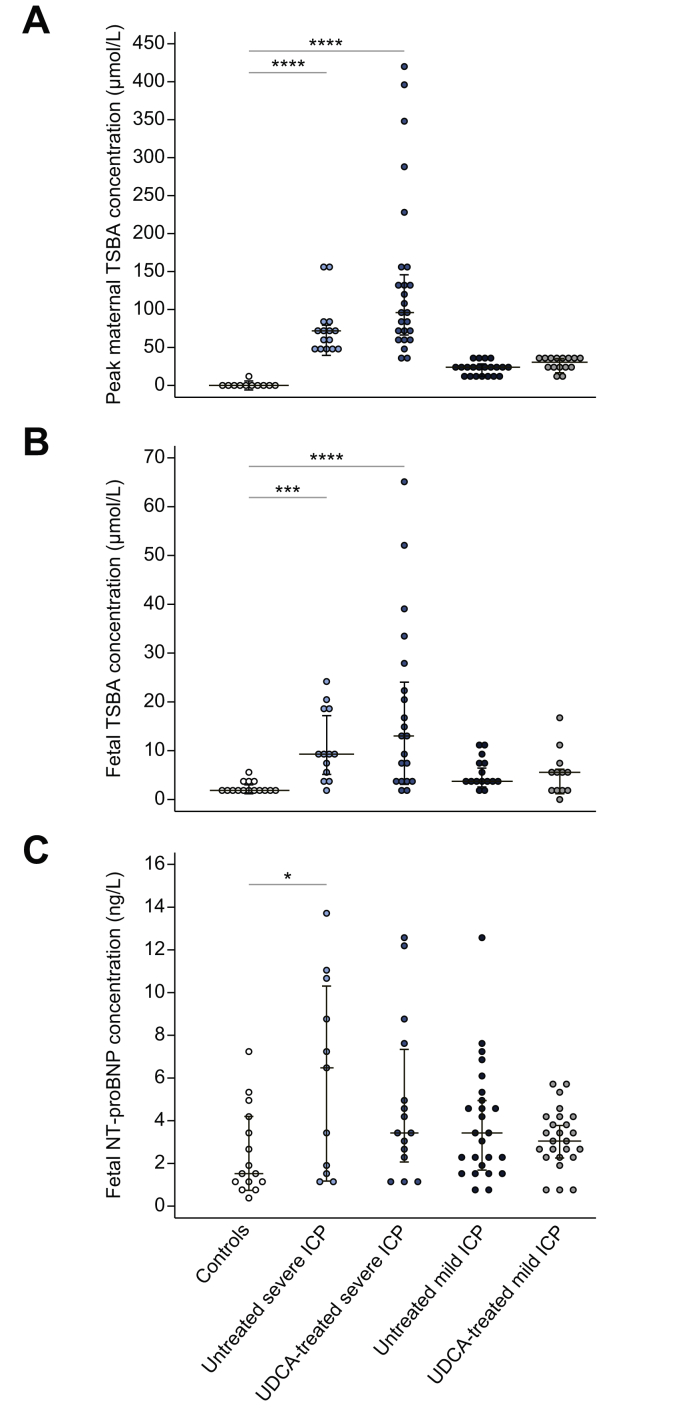
**Dot plots depicting TSBA and NT-proBNP concentrations.** Controls (n = 15), individuals with severe untreated ICP (n = 15), severe UDCA-treated ICP (n = 25), mild untreated ICP (n = 21) or UDCA-treated mild ICP (n = 15). (A) Peak maternal TSBA concentrations measured during pregnancy; (B) corresponding fetal TSBA concentrations measured using umbilical venous blood; (C) fetal NT-proBNP concentrations measured using umbilical venous blood. Analysis via Kruskal Wallis test with post-hoc Dunn's, *∗p* <0.05, ∗∗*∗p* <0.001, ∗∗∗*∗p* <0.0001. ICP, intrahepatic cholestasis of pregnancy; NT-proBNP, N-terminal pro-B-type natriuretic peptide; TSBA, total serum bile acid; UDCA, ursodeoxycholic acid.

One-way ANOVA demonstrated significant differences in peak maternal and fetal TSBA concentrations, and fetal NT-proBNP concentrations at delivery between participant cohorts (Fig. 2A-C). Multiple comparison tests indicated maternal and fetal TSBA concentrations in severe ICP were significantly higher than in controls (Fig. 2A and B). Similarly, fetal NT-proBNP concentrations were significantly higher in untreated severe ICP than in controls (Fig. 2C).

One-way ANOVA of individual bile acids in umbilical venous blood demonstrated significant differences in fetal TSBA and individual bile acid concentrations between cohorts (Table S3).

**Fig. 3:**
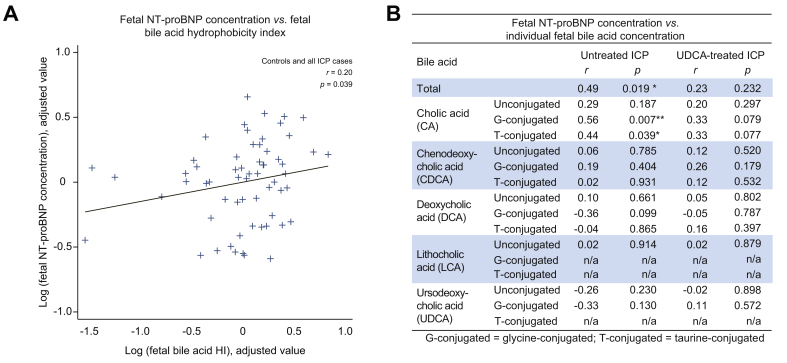
**Association between fetal bile acids and NT-proBNP concentrations**. (A) Added variable plot of partial correlation analysis between log-transformed fetal NT-proBNP concentration and fetal bile acid HI measured using umbilical venous blood samples taken from controls (n = 15), untreated ICP (n = 36) and UDCA-treated ICP (n = 40). (B) Table demonstrating partial correlation analysis of individual fetal serum bile acids and NT-proBNP concentrations from women with untreated (n = 36) or UDCA-treated (n = 40) ICP only. r = correlation coefficient. Significant *p* values are presented in bold. *∗p* <0.05, ∗*∗p* <0.01. HI, hydrophobicity index; ICP, intrahepatic cholestasis of pregnancy; NT-proBNP, N-terminal pro-B-type natriuretic peptide; UDCA, ursodeoxycholic acid.

There were significant positive correlations between fetal NT-proBNP and HI of the fetal bile acids (Fig. 3A) and with concentrations of fetal TSBA, glycocholic acid (GCA) and TCA in the untreated ICP cohort (Fig. 3B). No correlations were observed between fetal NT-proBNP concentrations and fetal TSBA in the UDCA-treated cohort (Fig. 3B).

### Maternal peak TSBA and ALT concentrations are positively correlated with fetal NT-proBNP concentrations

**Fig. 4:**
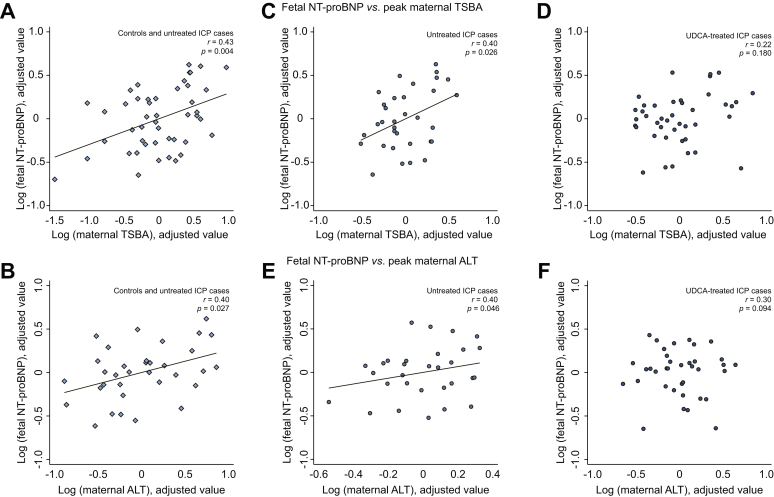
**Added variable plots of partial correlation analyses between fetal NT-proBNP and maternal TSBA or ALT concentrations**. (A & D) In both controls (n = 15) and women with untreated ICP (n = 36); (B & E) in women with untreated ICP only (n = 36); (C & F) in women with UDCA-treated ICP only (n = 40). Plots with both controls and cases on the same graph have diamond-shaped data points, plots with untreated ICP cases only have circle-shaped shaded data points and plots with UDCA-treated cases only have circle-shaped hollow data points. ALT, alanine aminotransferase; ICP, intrahepatic cholestasis of pregnancy; NT-proBNP, N-terminal pro-B-type natriuretic peptide; TSBA, total serum bile acid; UDCA, ursodeoxycholic acid.

A significant positive partial correlation was observed between fetal NT-proBNP and maternal concentrations of TSBA and ALT when analyzing untreated ICP cases and controls together (Fig. 4A and B). No correlation was found between fetal NT-proBNP and maternal bilirubin concentrations (Fig. S1). Analysis of untreated ICP cases demonstrated significant positive correlations between fetal NT-proBNP and maternal TSBA and ALT concentrations (Fig. 4C and E). No correlations were observed in the UDCA-treated group (Fig. 4D and F).

### Maternal TSBA concentrations are positively correlated with the prolongation of the fetal PR interval length, but not the length of the QTc interval or QRS duration

**Fig. 5:**
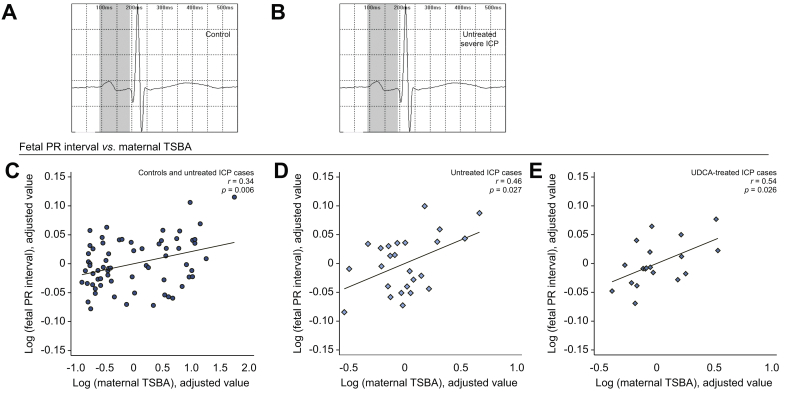
**Association between ICP and PR interval prolongation** (A & B) Representative averaged fECG waveforms demonstrating PR interval prolongation (grey) in untreated severe ICP (C) added variable plot demonstrating the partial correlation between maternal TSBA concentration and fetal PR interval length in both controls (n = 43) and women with untreated ICP (n = 26), (D) in women with untreated ICP only (n = 26), (E) in women with UDCA-treated ICP only (n = 22). Plots with both controls and cases have circle-shaped data points, plots with untreated ICP cases only have diamond-shaped shaded data points and plots with UDCA-treated cases only have diamond-shaped hollow data points. fECG, fetal electrocardiogram; ICP, intrahepatic cholestasis of pregnancy; TSBA, total serum bile acid; UDCA, ursodeoxycholic acid.

One-way ANOVA demonstrated significant differences in fetal PR interval length between cohorts (Table S4). Partial correlation analysis demonstrated a significant positive correlation between maternal TSBA and fetal PR interval length measurements when analyzing untreated ICP cases and controls together (Fig. 5C). Analysis of ICP cases demonstrated significant positive correlations between maternal TSBA concentrations and fetal PR interval length for both untreated and UDCA-treated cases (Fig. 5D and E, respectively). No significant correlations with maternal TSBA concentration were observed when analyzing fetal QTc interval length or QRS duration (Fig. S2A and B, respectively). There were no significant correlations between maternal TSBA concentrations and maternal CTI (Fig. S3A-C).

### Maternal TSBA concentrations positively correlated with fetal heart rate variability

To investigate ICP-induced changes in fHRV, we performed time-domain analysis on the RR intervals derived from fECGs of women with untreated ICP. One-way ANOVA demonstrated significant differences in root mean square of successive differences (RMSSD) and standard deviation of normal to normal intervals (SDNN) values in behavioral state 2F (Table S4).

**Fig. 6:**
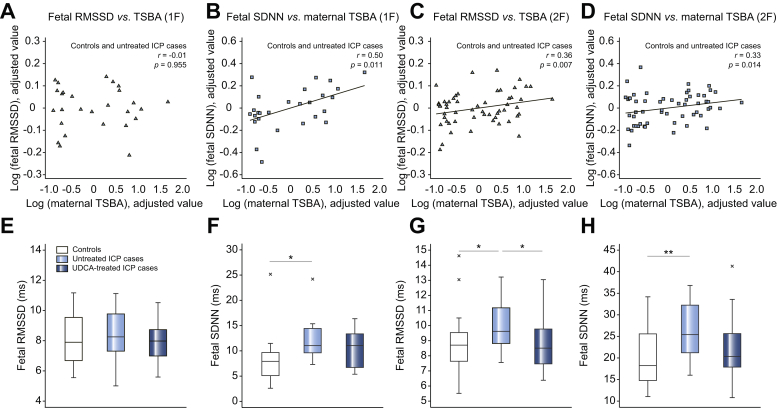
**Association between untreated ICP and fetal heart rate variability** (A-D) Added variable plots demonstrating the partial correlation between maternal TSBA concentration and fetal heart rate variability measurements in controls (n = 16 in 1F and n = 33 in 2F) and untreated ICP (n = 13 in 1F and n = 36 in 2F). RMSSD data points are triangles whilst SDNN data points are squares. (E-H) Box and whisker plots demonstrating the medians and IQRs of fetal heart rate variability measurements of controls (n = 16 in 1F and n = 33 in 2F), untreated ICP (n = 13 in 1F and n = 36 in 2F) and UDCA-treated ICP (n = 15 in 1F and n = 20 in 2F). Analysis via Kruskal Wallis test with post-hoc Dunn's. *∗p* <0.05, ∗*∗p* <0.01. ICP, intrahepatic cholestasis of pregnancy; RMSSD, root mean square of successive differences; SDNN, standard deviation of normal to normal intervals; TSBA, total serum bile acid; UDCA, ursodeoxycholic acid.

Partial correlation analysis demonstrated a lack of correlation between maternal TSBA concentrations and fetal RMSSD values from behavioral state 1F (quiet sleep), while a significant positive correlation was observed in values from behavioral state 2F (active sleep) (Fig. 6A and C, respectively). Positive correlations were also observed between maternal TSBA concentrations and fetal SDNN values in both behavioral states (Fig. 6B and D, respectively).

When all untreated cases were compared using one-way ANOVA, there was a significant increase of RMSSD values in behavioral state 2F (active sleep) (9.6 [8.8–11.3] *vs.* 8.7 [7.6–9.6] ms, *p =* 0.028, Fig. 6G) and SDNN values in behavioral state 1F (quiet sleep) (11.0 [9.5–14.9] *vs.* 7.9 [5.1–9.7] ms, *p =* 0.013, Fig. 6F) and active sleep 2F (25.4 [21.0–32.4] *vs.* 18.2 [14.7–25.7] ms, *p =* 0.003, Fig. 6H). UDCA treatment was associated with a significant reduction in RMSSD in behavioral state 2F cases (8.5 [7.4–9.7] ms, *p =* 0.030, Fig. 6G), and there was a non-significant attenuation of the ICP-associated increase in SDNN values in UDCA-treated cases (Fig. 6F and H). There were no significant correlations between maternal TSBA concentrations and maternal HRV values (Fig. S3D-F).

## Discussion

In this study, we have demonstrated that untreated ICP is associated with a fetal cardiac phenotype that positively correlates with fetal and maternal serum bile acid concentrations (summarized in Table S5). We found that the fetuses of women taking UDCA treatment did not have the same dysfunctional cardiac phenotype for most parameters, suggesting that UDCA may be cardioprotective.

The fetal cardiac phenotype observed in untreated ICP constituted, in part, an elevated fetal NT-proBNP concentration in umbilical venous blood, which is an indicator of fetal distress, tachy- and bradyarrhythmia and heart failure in fetuses with congenital heart defects.^35^ The range of NT-proBNP concentrations obtained from control participants was similar to previous reports, therefore elevated NT-proBNP concentrations suggest that ICP fetuses have a cardiac phenotype concordant with arrhythmic activity and fetal distress.^36^

Measurement of total and individual serum bile acids in umbilical venous blood demonstrated that fetal TSBA, GCA and TCA concentrations and bile acid HI are positively correlated with fetal NT-proBNP concentrations. This is the first study to report this association in untreated ICP. GCA and TCA form the majority of the maternal serum bile acid pool in ICP.^37^ CA and TCA administration causes cardiac dysfunction in experimental models and both are demonstrably deleterious in comparison to UDCA.[29], [30], [31]^,^^38^ This suggests that fetal cardiotoxicity may be an explanation for the novel finding of a correlation between elevated NT-proBNP concentration and increased fetal bile acid HI, an established indicator of bile acid cytotoxicity.^27^

The association of the severity of fetal cardiac phenotype with increasing serum concentrations of maternal and/or fetal TSBA is in agreement with previous studies.^11^^,^^14^^,^^15^^,^^19^ The severity of hypercholanemia is associated with adverse fetal outcomes, *e.g.* stillbirth when maternal TSBA concentrations exceed 40 μmol/L and markedly so at ≥100 μmol/L.[2], [3], [4] No stillbirth events occurred in our study, although only 3 participants had TSBA concentrations of ≥100 μmol/L; 1 untreated fECG study participant had a TSBA concentration of 187 μmol/L and 2 untreated NT-proBNP assay participants had TSBA concentrations of 100 μmol/L and 151 μmol/L.

UDCA-treated participants did not have the same fetal cardiac phenotype as those with untreated ICP. Although the association between maternal TSBA concentration and PR interval prolongation remained in this cohort, there were no longer associations between maternal or fetal serum bile acid concentrations and fHRV parameters or NT-proBNP concentrations. Lack of association between bile acid concentrations and cardiac parameters in the UDCA-treated cohort may be explained by alterations in the fetal bile acid composition and HI, which have previously been demonstrated in studies of both healthy and hypercholanemic patients.^48^^,^^49^ The bile acid HI may be of more relevance than the TSBA concentration, as it is notable that the TSBA concentrations were not lower in the maternal or fetal serum samples following UDCA treatment. Consistent with this observation, UDCA treatment alters the concentration of individual bile acids in maternal serum; specifically, the proportion of UDCA rises from <0.5% to 60% whilst CA reduces from 51% to <20% in the serum of women with ICP after UDCA treatment.^50^ Unlike untreated participants, fetal TSBA and GCA concentrations in UDCA-treated participants did not correlate with fetal NT-proBNP concentrations, which is in line with the calculated HI and their association with fetal NT-proBNP concentration.

The recent PITCHES trial did not demonstrate an impact of UDCA treatment on a composite measure of adverse pregnancy outcome that included stillbirth, preterm birth and neonatal unit admission.^25^ This is not contradictory to our data related to an ICP-associated fetal cardiac phenotype as this study of fECG is of particular relevance to the etiology of stillbirth. There were 3 stillbirths in the PITCHES trial cohort (2 did not receive UDCA treatment) from a total of 604 participants.^25^ Furthermore, 76% of the 605 PITCHES participants had TSBA concentrations <40 μmol/L, and only 7% participants had concentrations ≥100 μmol/L at the time of randomization.^25^

The positive correlation between maternal TSBA concentrations and fetal PR interval length is in agreement with previous studies, although it may not have clinical significance as the majority of participants had PR interval lengths below established thresholds for screening of first-degree atrioventricular (AV) block.^39^ Experimental data has demonstrated that bile acid-induced PR interval prolongation occurs via TCA activation of muscarinic acetylcholine M_2_ receptors, resulting in intracellular calcium dynamic disruption via T-type voltage-dependent calcium channels and slowing of conduction velocity at the AV node.^29^^,^^44^ This suggests a mechanism by which higher circulating TCA concentrations cause fetal PR interval length prolongation in untreated ICP.

Fetal PR interval prolongation has previously been reported in both untreated and UDCA-treated cohorts, suggesting that UDCA treatment is not completely protective.^18^ PR interval prolongation was observed in our UDCA-treated cohorts; however, individual bile acids were not measured in maternal serum of these participants and therefore responsiveness to UDCA is unknown. UDCA has a cardioprotective effect in experimental fetal heart models.[28], [29], [30], [31] TCA-induced PR interval prolongation observed in an *in vitro* model of the fetal heart was attenuated by UDCA, although 400 μM of TCA was used which is rarely physiologically observed in ICP and suggests that UDCA may only be protective in a subset of women with markedly elevated TSBA concentrations.^29^ UDCA hyperpolarizes the membrane voltage of *in vitro* myofibroblasts via the sulphonylurea receptors, thereby increasing potassium conductance and counteracting the effects of TCA.^31^ It also acts as an agonist for the bile acid receptor Tgr5 (GPBAR1) in murine ventricular cardiomyocytes although it did not elicit contractile changes in this cell type.^41^ Cardioprotective effects of UDCA in non-cholestatic adults with cardiac disease have also been reported and serum concentrations of conjugated UDCA are lower in patients who have had atrial fibrillation events.^28^^,^^42^^,^^43^

The lack of association between maternal TSBA concentrations and fetal QTc interval length is also in agreement with previous studies.^40^ Our data has shown a lack of correlation between maternal TSBA concentrations and maternal ECG parameters in comparison to control pregnancies. Maternal QTc interval lengths were found to be within normal range which is contrary to a previous study using an adult 12-lead ECG device.^20^ Ideally, diurnal post-prandial fluctuations in TSBA concentrations and their relationship with fetal cardiac dysfunction would also be evaluated, but the requirement for hourly venepuncture from pregnant women and the inability to obtain good quality fECG data during wakefulness precluded this approach.

The behavioral state of the fetus is indicative of fetal wellbeing and is closely associated with fetal movement, FHR and fHRV.^21^ This is the first study to our knowledge that investigates behavioral state-specific time-domain measurements of fHRV in women with untreated and UDCA-treated ICP. Statistically significant differences were more prominent in behavioral state 2F (active sleep), likely due to fetuses spending more time in this behavioral state, resulting in a larger number of data points, as well as the wider scope of fHRV fluctuation when the fetus is in an active state.^22^ Median SDNN and RMSSD values from case cohorts were within the normal range cited in previous literature and no infants in our study had clinical indicators of fetal hypoxia at delivery, although there are reports of ICP-induced fetal hypoxia in other cohorts.[45], [46], [47]

A limitation of this study is the fact that the UDCA-treated and untreated participants were not matched for severity of hypercholanemia. In future, if it were feasible to study a prospectively recruited cohort of untreated *vs.* UDCA-treated women with TSBA concentrations of ≥100 μmol/L, one could confirm the association between fetal cardiac function and highly elevated TSBA concentrations. The optimal experimental design would have taken both umbilical venous blood and fECG samples from the same participants to avoid differences in demographic data between severe ICP cohorts. Additionally, data on the duration of delivery would also be collected as part of multivariate statistical analysis of fetal NT-proBNP concentrations. To eliminate the potential effect of individual differences in HRV, this study should be repeated with a larger cohort of ICP participants prior to and after UDCA treatment with responsiveness to the drug also assessed.

### Conclusion

This study has demonstrated a fetal cardiac phenotype that is associated with severity of ICP in untreated pregnancies. Although further investigation is required to assess the impact and importance of these anomalies, these data provide a step towards understanding the mechanisms of ICP-associated fetal cardiac pathologies and support the hypothesis that elevated circulating fetal bile acids may predispose the fetal heart to a sudden arrhythmic event. Further studies measuring fetal cardiac parameters in a larger cohort, particularly in women who have serum bile acid concentrations ≥100 μmol/L, are required to establish the risk of elevated serum bile acid concentrations, to stratify those who would benefit most from increased fetal heart monitoring, and to clarify whether UDCA treatment is protective for these high-risk pregnancies.

### Abbreviations

CA, cholic acid; CDCA, chenodeoxycholic acid; CTG, cardiotocography; CTI, cardiac time interval; DCA, deoxycholic acid; ECG, electrocardiogram; fECG, fetal electrocardiogram; FHR, fetal heart rate; GCA, glycocholic acid; fHRV, fetal heart rate variability; HI, hydrophobicity index; HRV, heart rate variability; ICP, intrahepatic cholestasis of pregnancy; NT-proBNP, N-terminal pro-B-type natriuretic peptide; RMSSD, root mean square of successive differences; SDNN, standard deviation of normal to normal intervals; TCA, taurocholic acid; TSBAs, total serum bile acid; UDCA, ursodeoxycholic acid.

## Financial support

The study was funded by the 10.13039/100010269Wellcome Trust (Programme Grant 092993/Z/10/Z), Tommy's Charity, The John Coates Charitable Trust, the 10.13039/501100000272National Institute for Health Research Biomedical Research Centres at 10.13039/501100004941Guy's and St Thomas' NHS Foundation Trust (GSTFT) and 10.13039/501100000764King's College London (IS-BRC-1215-20006) and 10.13039/501100000762Imperial College Healthcare NHS Trust (ICHT), 10.13039/100011042ICP Support, United Kingdom and the 10.13039/501100001922NIHR Efficacy and Mechanism Evaluation Programme. CW is funded by an NIHR Senior Investigator award and LCC has an NIHR Professorship. IAJ was on supported study leave from the 10.13039/501100009826University of Colombo, Sri Lanka. JG is funded by 10.13039/501100000327Heart Research UK. Monica Healthcare Limited provided support and advice. The views expressed are those of the authors and not necessarily those of the NHS, NIHR or the Department of Health. We thank the Research Midwives and Trial Coordinators at GSTFT, ICHT and St. Richard’s Hospital.

## Authors’ contributions

The final study design was conceived by TV and CW based on initial studies by JT, IAJ, FBP and PL. Ethical approval applications for this study were obtained by VG, TV and JC. The manuscript was written by TV and CW. Umbilical venous blood was collected by research staff at GSTFT and ICHT. NT-proBNP concentrations were assayed by TV. UPLC-MS/MS was conducted by ALS and HUM; results were analyzed by TV. ECGs were collected by research staff at GSTFT, ICHT and SRH as well as IAJ, SD and TV. MATLAB scripts for HRV analysis were provided by ML, JY and WPF. Processing of ECG data was conducted by TV. Analysis of ECG data was conducted by TV, SD and CM. Statistical advice was provided by PS; statistical analysis was conducted by TV. All authors contributed to reviewing the manuscript.

## Data availability statement

Participants who gave samples or data to this study provided informed consent on the basis that anonymised data would only be available authorised members of the study team; sharing of anonymised data is only permitted to research groups who are investigating specific pregnancy-related conditions.

## Conflict of interest

BHG has previously served as a director for Monica Healthcare Limited and has no commercial or financial connections in the company. CW and HUM are consultants with Mirum Pharmaceuticals and CW is a consultant for GlaxoSmithKline. The remaining authors have no conflicts of interest to disclose.

Please refer to the accompanying ICMJE disclosure forms for further details.

## Supplementary data

Supplementary information.pdf

CTAT table.pdf
