# Supplementary material for: Fetal cardiac dysfunction in intrahepatic cholestasis of pregnancy is associated with elevated serum bile acid concentrations
Source: J Hepatol. 2021 May;74(5):1087–96. doi: 10.1016/j.jhep.2020.11.038 (PMC8062912; doi:10.1016/j.jhep.2020.11.038)
Supplement: Supplementary information.pdf [file mmc1.pdf]

Fetal cardiac dysfunction in intrahepatic cholestasis of pregnancy is associated with elevated serum bile acid concentrations

Tharni Vasavan, Sahil Deepak, Indu Asanka Jayawardane, Maristella Lucchini, Catherine Martin, Victoria Geenes, Joel Yang, Anita Lövgren-Sandblom, Paul Townsend Seed, Jenny Chambers, Sophia Stone, Lesia Kurlak, Peter Hendy Dixon, Hanns-Ulrich Marschall, Julia Gorelik, Lucy Chappell, Pam Loughna, Jim Thornton, Fiona Broughton Pipkin, Barrie Hayes-Gill, William Paul Fifer, Catherine Williamson

Table of contents

Supplementary methods 2

Supplementary figure legends 6

Fig. S1 8

Fig. S2 9

Fig. S3 10

Table S1 10

Table S2 12

Table S3 13

Table S4 14

Table S5 15

References 16

Supplementary methods

Recruitment for ECG recording

Women were not eligible for recruitment if they had a multifetal pregnancy, were in active labour, had diabetes mellitus, hypertension or any cardiac/liver disorders or if the fetus had identified congenital abnormalities. Birth weight centile for fetuses who underwent ECG and umbilical venous blood sample collection was calculated using GROW software and based on the mean birth weight reported in 2012 in England and Wales [1].

Based on questionnaires submitted after ECG recordings, no women felt unwell or had decreased fetal movements at the time of recording or up to 24 hours afterwards.

ECG data processing

Monica DK v1.9 software (Monica Healthcare Limited, Nottingham UK) was used to obtain a trace of fetal heart rate (FHR), maternal heart rate (MHR) and maternal movement over the recording period. In order to remove probably periods of maternal wakefulness, the first and last hour of the recording were excluded from analysis. A two-hour period of the recording was chosen where there was a low indication of maternal movement through observation of the Monica AN24 inbuilt accelerometer and acceleration patterns in the MHR as well as ensuring the chosen period had a minimal loss of FHR signal. This 2-hour period was used for PR, QT interval length measurement and heart rate variation (HRV) analysis.

Analysis of cardiac time intervals

The PR and QT intervals were measured by two independent observers who were familiar with ECG waveform analysis. Monica DK v1.9 was used to extract the fetal or maternal

ECG waveform from the raw recordings (sample frequency 1kHz), via an inbuilt algorithm that detects R peak frequency and amplitude. Monica DK v1.9 was used to create an averaged ECG waveform of all identified cardiac cycles that lay within a specified heart rate range in the two-hour period. An averaged waveform created from the FHRs of 110 to 169 bpm was used to mark cardiac time intervals in the fetus. The P, Q, R, S and T of each averaged ECG waveform were marked and the PR and QT interval automatically calculated by the Monica DK v1.9. Analysis of the maternal ECG utilised the same two-hour period of the recording. ECG data that did not have a clearly identifiable P and T wave were excluded from analysis; all other morphological abnormalities were also noted. QT intervals were corrected for FHR using Bazett's formula.

Fetal behavioural state coding

MATLAB vR2017a software (Mathworks Inc, US) was used to plot the FHR in beats per minute (bpm) (sampled at 0.25s intervals) vs. time in the selected two-hour analysis period. The behavioural state of the fetus was assessed prior to HRV analysis in order to ensure equivalent behavioural states were compared during statistical analysis of HRV. Behavioural state, classed as 1F (quiet sleep), 2F (active sleep), 3F (quiet awake), 4F (active awake) or "indeterminable" was allocated for the entirety of the selected two-hour analysis period as previously described; each determinable state was required to have a minimum duration of 3 minutes [3].

HRV analysis

Time-domain HRV analysis was conducted on the fetal and maternal ECG files via the calculation of the root mean squared of successive differences (RMSSD) and standard

deviation of normal to normal intervals (SDNN) and median heart rate. A .csv file containing maternal and fetal R-R intervals was extracted from the raw ECG file using Monica DK v1.9. MATLAB was used to assess the quality of the recording per 30 second epoch based on a standard length and absolute difference between consecutive intervals [4]. Epochs which were ≤70% quality within the chosen two-hour window were excluded from analysis. The mean R-R interval value of each 30 second epoch was determined using MATLAB; these values were subsequently used to calculate the median RMSSD and SDNN after fetal behavioural state for each epoch was allocated using the method described above.

Statistical Analyses

Missing delivery data is indicated as "unknown" in Supplementary Tables 1 and 2; these data were excluded from statistical analyses. Due to the low numbers of data available for neonatal unit admission, this covariate was omitted from all correlation analyses. Information on maternal bilirubin concentrations of participants who underwent ECG analysis was not collected due to the lack of association with bilirubin in the previous umbilical venous blood assays. Correlation analyses between maternal ALT concentrations and fetal cardiac parameters were not conducted due to a lack of a significant difference in concentration between groups as measured by Kruskal-Wallis ANOVA.

Added variable scatter plots of the aforementioned partial correlation analyses are presented with adjusted data points based on covariates; fitted lines have been drawn in plots that reported statistically significant correlation coefficients (r). Partial correlation analysis was initially conducted between control and untreated cases as one cohort to establish the impact of a range of TSBA concentrations on parameters of interest. Subsequent analyses investigated only untreated and UDCA-treated ICP cases if the initial correlation was significant. Due to low numbers of cases for the HRV analysis, Kruskal Wallis ANOVA with

post-hoc Dunn's test was conducted between case and control cohorts instead of a separate partial correlation to assess the effect of UDCA treatment. Individual dot plots and box and whisker plots have been presented to report the results of these analyses.

Fig. Slegends

Fig. S1: Lack of association between maternal bilirubin and fetal NT-pro-BNP concentration. Added variable plot demonstrating the lack of partial correlation between fetal NT-proBNP and peak maternal bilirubin concentrations in control participants (n=15) and participants with untreated ICP (n=36).

Fig. S2: Lack of association between maternal TSBA concentration and fetal QTc and QRS lengths (A) Added variable plot demonstrating the lack of partial correlation between fetal QTc interval length and maternal TSBA concentrations in control participants (n=43) and participants with untreated ICP (n=26) (B) Added variable plot demonstrating the lack of partial correlation between fetal QRS duration and maternal TSBA concentrations in control participants (n=43) and participants with untreated ICP (n=26).

Fig. S3: Lack of association between maternal TSBA concentration and ECG parameters (A-C) Added variable plots demonstrating the lack of correlation between maternal TSBA concentration and maternal cardiac time interval measurements of controls (n = 43) and participants with untreated ICP (n = 26) (D-F) Added variable plots demonstrating the lack of correlation between maternal TSBA concentration and maternal heart rate variability measurements of controls (n = 43) and participants with untreated ICP (n = 26)

Table S1: Demographic and delivery details of the participants from whom umbilical venous blood was collected for NT-proBNP measurement and UPLC-MS/MS measurement of bile acid profiles. Significant differences between all cohorts as measured

by Kruskal-Wallis ANOVA have been reported without additional multiple comparison tests between pairs of cohorts. Results are presented as median [IQR] or n (%). Significant p values are presented in bold. *=p<0.05, **=p<0.005, ***=p<0.0005.

Table S2: Demographic and delivery details of the participants who underwent ECG recording and analysis. Significant differences between cohorts have been analysed via Kruskal-Wallis ANOVA without multiple comparison tests. Results are presented as median [IQR] or n (%). Significant p values are presented in bold. *=p<0.05, ***=p<0.0005.

Table S3: Fetal and maternal laboratory and treatment details of the participants from whom umbilical venous blood was collected for NT-proBNP measurement. Results are presented as median [IQR] or n (%). Significant p values are presented in bold. *=p<0.05, **=p<0.005, ***=p<0.0005, ****=p<0.0001. ND signifies "not detected".

Table S4: Laboratory details, cardiac time interval measurements and heart rate variability measurements of participants who underwent ECG recording and analysis. Results are presented as median [IQR]. Significant p values are presented in bold. *=p<0.05, ***=p<0.0005.

Table S5: Table summarising the main findings and associations observed between biochemical and fetal cardiac parameters in this study.

Fig. S1

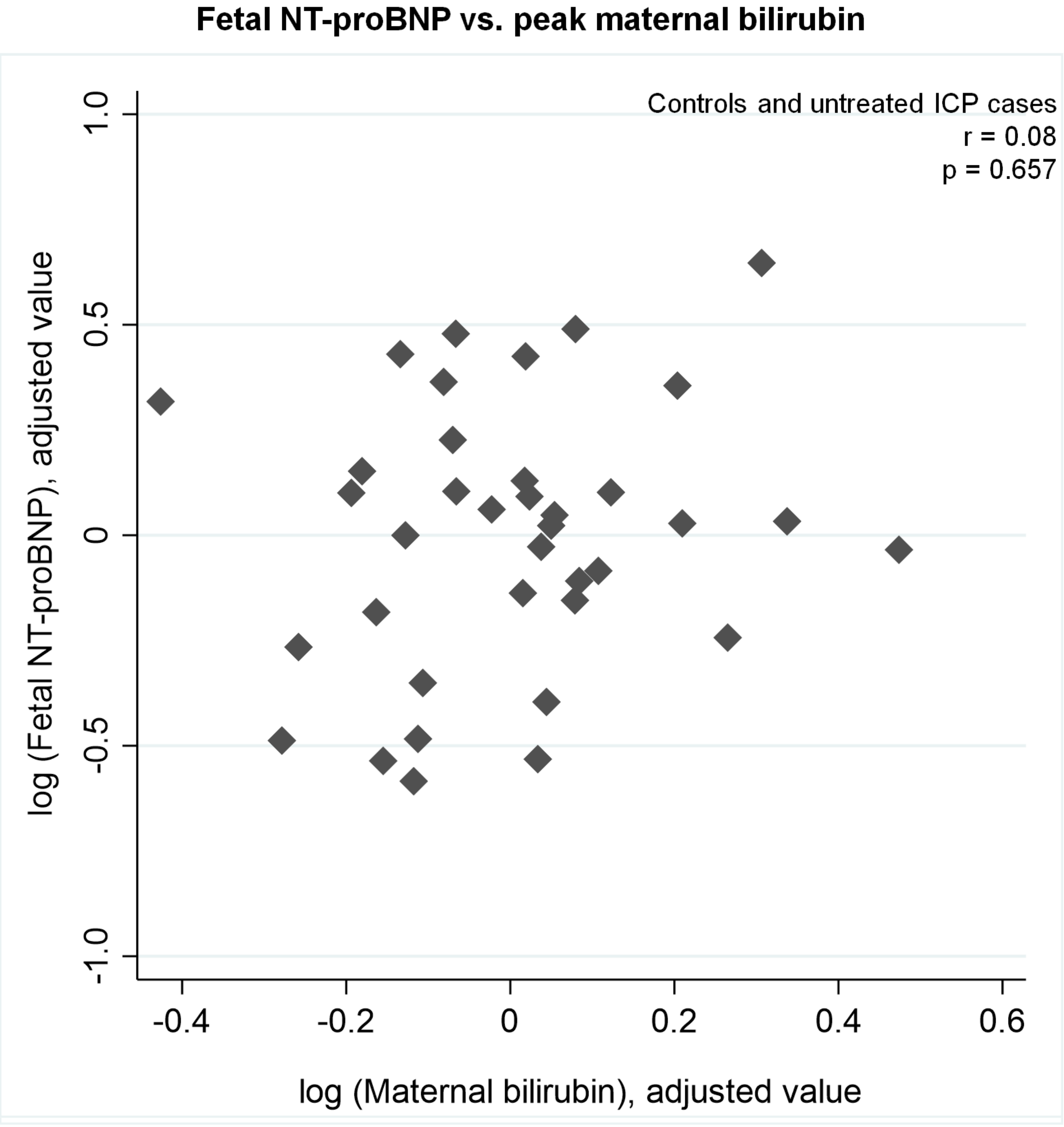
Fetal NT-proBNP vs. peak maternal bilirubin
Controls and untreated ICP cases
r = 0.08
p = 0.657
log (Fetal NT-proBNP), adjusted value
1.0
0.5
0
-0.5
-1.0
-0.4
-0.2
0
0.2
0.4
0.6
log (Maternal bilirubin), adjusted value

Fig. S2

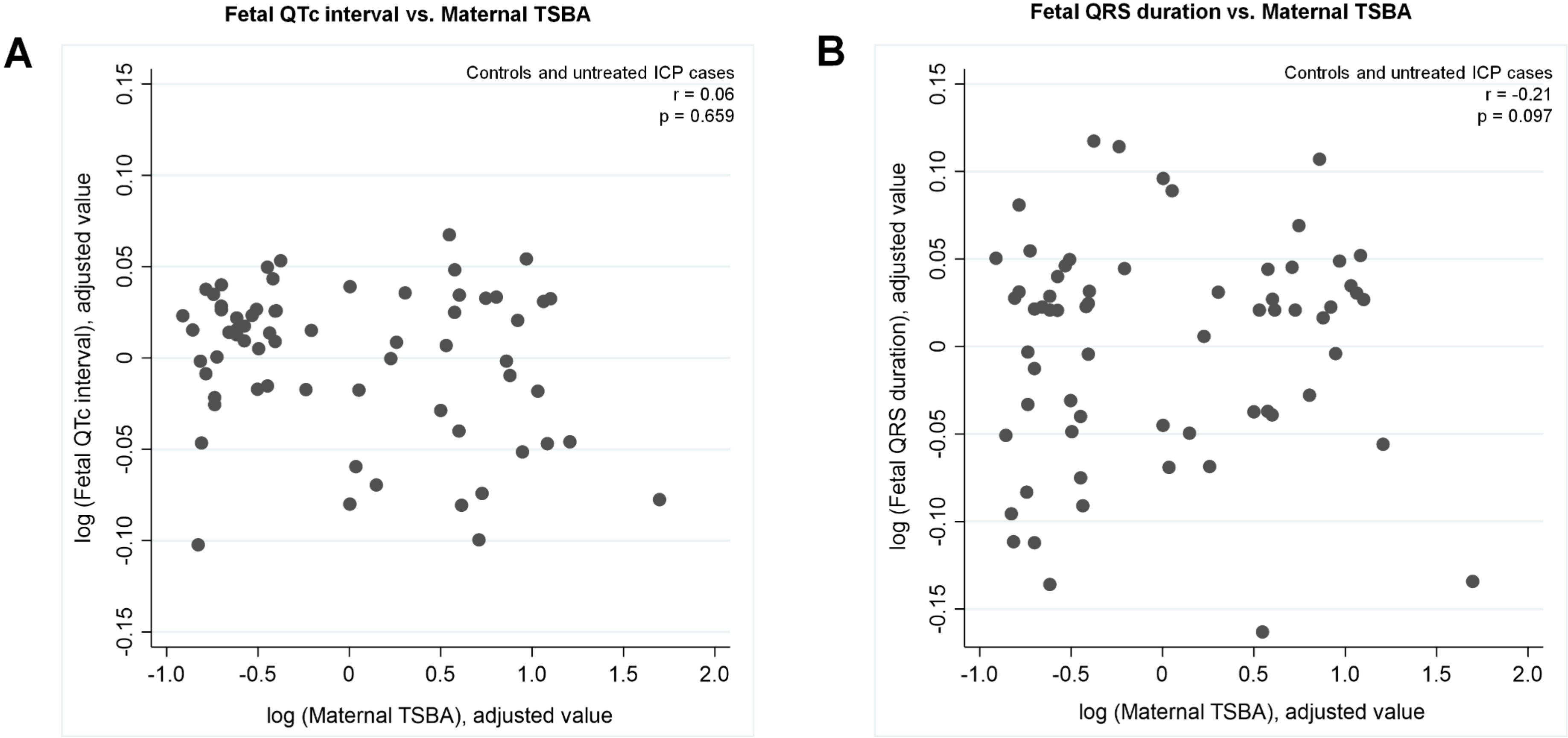
Fetal QTc interval vs. Maternal TSBA
A
Controls and untreated ICP cases
r = 0.06
p = 0.659
log (Fetal QTc interval), adjusted value
log (Maternal TSBA), adjusted value
-0.15
-0.10
-0.05
0
0.05
0.10
0.15
-1.0
-0.5
0
0.5
1.0
1.5
2.0
Fetal QRS duration vs. Maternal TSBA
B
Controls and untreated ICP cases
r = -0.21
p = 0.097
log (Fetal QRS duration), adjusted value
log (Maternal TSBA), adjusted value

Fig. S3

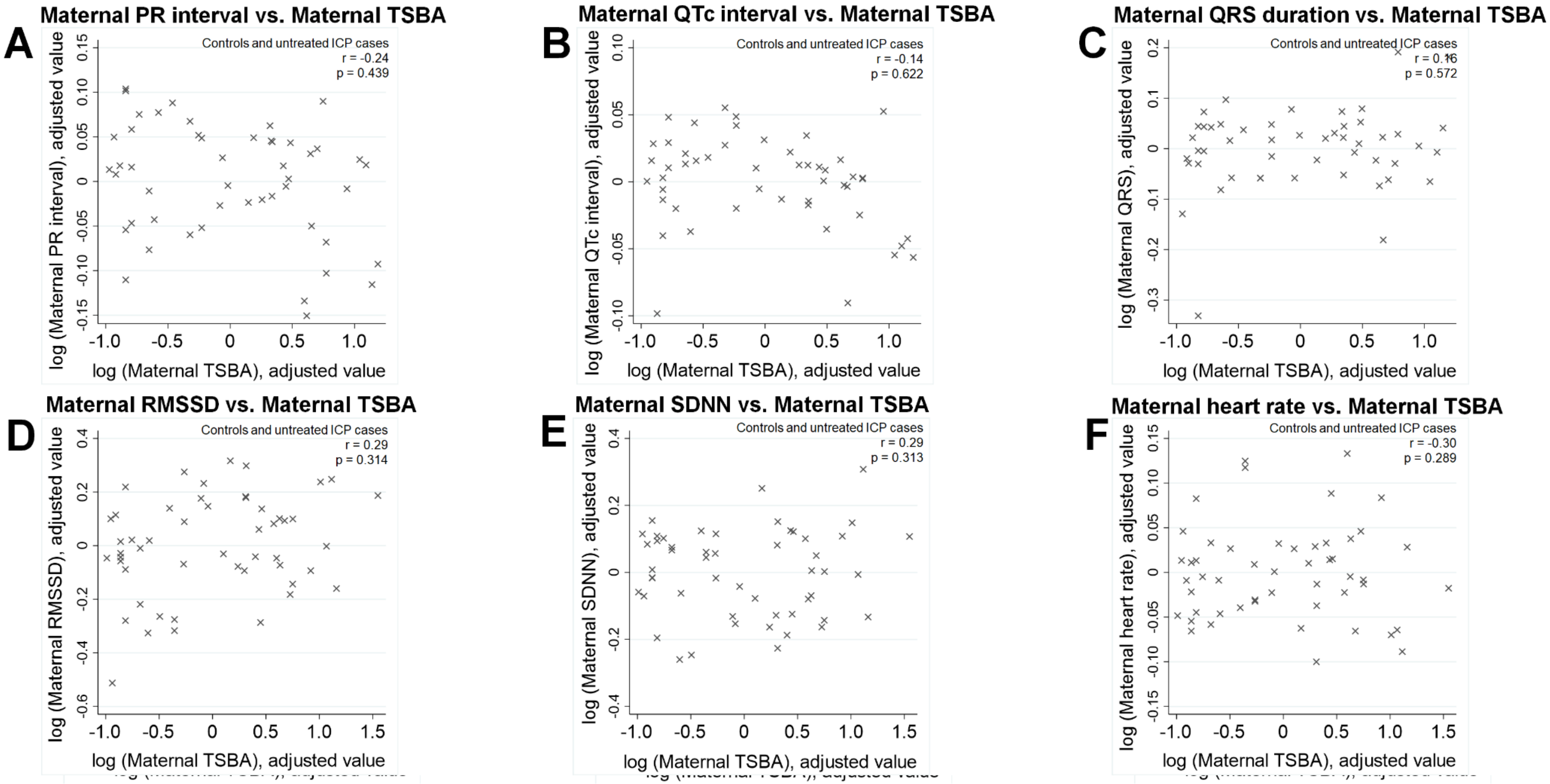

Table S1

		Control	Untreated Severe ICP	UDCA-treated Severe ICP	Untreated Mild ICP	UDCA-treated Mild ICP	*p*
**n**		**15**	**15**	**25**	**21**	**15**	
**Maternal ethnicity (n)**	White British	7 (47)	6 (40)	15 (60)	6 (29)	6 (40)	*0.258*
	White other	3 (20)	6 (40)	6 (24)	11 (52)	3 (20)	
	Asian or Asian other	3 (20)	2 (13)	2 (8)	1 (5)	1 (7)	
	Black or Black other	1 (7)	1 (7)	0 (0)	3 (14)	3 (20)	
	Other / Unknown	1 (7)	0 (0)	2 (8)	0 (0)	2 (13)	
**Induction of labour (n)**	Yes	0 (0)	9 (60)	13 (52)	18 (86)	11 (73)	*<0.001 ****
	No	10 (67)	5 (33)	11 (44)	2 (10)	4 (27)	
	Unknown	5 (33)	1 (7)	1 (4)	1 (5)	0 (0)	
**Mode of delivery (n)**	SVD	1 (7)	9 (60)	11 (44)	11 (52)	9 (60)	***0.011 ****
	ELCS	1 (7)	1 (7)	3 (12)	6 (29)	3 (20)	
	EMCS	9 (60)	4 (27)	9 (36)	1 (5)	1 (7)	
	Instrumental	2 (13)	1 (7)	2 (8)	2 (10)	2 (13)	
	Unknown	2 (13)	0 (0)	0 (0)	1 (5)	0 (0)	
**Sex (n)**	Female	8 (53)	5 (33)	15 (60)	8 (38)	5 (33)	*0.270*
	Male	5 (33)	10 (67)	10 (40)	11 (52)	10 (67)	
	Unknown	2 (13)	0 (0)	0 (0)	2 (10)	0 (0)	
**Gestational age at delivery (weeks^{+days})**		39^{+2} [39^{+0},40^{+2}]	38^{+0} [37^{+1},38^{+2}]	37^{+1} [35^{+6},37^{+4}]	38^{+2} [38^{+1},39^{+4}]	38^{+0} [37^{+3},38^{+5}]	***0.0001 *****
**Birth weight (g)**		3948 [3495,4330]	3302 [3050,3402]	3070 [2428,3308]	3270 [2975,3468]	3340 [3050,3645]	***0.0001 *****
**Birth weight centile (%)**		93.0 [75.3,100.0]	79.0 [49.0,98.0]	69.0 [26.0,94.8]	57.0 [36.0,90.8]	77.0 [56.0,94.0]	*0.109*
**APGAR 1 minute ≤ 7 (n)**		1 (7)	2 (13)	2 (8)	3 (14)	0 (0)	*n/a*
**APGAR 5 minutes ≤ 7 (n)**		0 (0)	0 (0)	1 (4)	0 (0)	0 (0)	*n/a*
**Arterial pH ≤ 7.0 (n)**		0 (0)	0 (0)	0 (0)	0 (0)	0 (0)	*n/a*
**Venous pH ≤ 7.0 (n)**		0 (0)	0 (0)	0 (0)	0 (0)	0 (0)	*n/a*
**Non-reassuring CTG during labour (n)**		0 (0)	4 (27)	1 (4)	5 (24)	6 (40)	*0.661*
**Presence of meconium-stained amniotic fluid (n)**		1 (7)	3 (20)	4 (16)	2 (10)	0 (0)	*0.309*
**Admission to NNU (n)**		0 (0)	1 (7)	6 (24)	0 (0)	0 (0)	***0.006 *****

Table S2

		Control	Untreated Severe ICP	UDCA-treated Severe ICP	Untreated Mild ICP	UDCA-treated Mild ICP	*p*
**n**		**43**	**10**	**6**	**16**	**16**	
**Maternal age (years)**		34.0 [30.5,36.0]	37.0 [32.0, 39.0]	30.5 [26.0, 35.0]	36.0 [26.0,37.0]	33.5 [30.3,39.8]	*0.720*
**Gestatational age at recording (weeks^{+days})**		34^{+0} [30^{+2},37^{+0}]	35^{+0}, [31^{+6}, 36^{+6}]	33^{+5} [28^{+0},36^{+2}]	35^{+0} [34^{+5},36^{+3}]	35^{+5} [34^{+6},36^{+5}]	*0.433*
**Maternal ethnicity (n)**	White British	26 (59.1)	5 (50.0)	5 (83.3)	9 (56.3)	10 (62.5)	*0.203*
	White other	9 (20.5)	1 (10.0)	1 (16.6)	4 (25.0)	5 (31.3)	
	Asian or Asian other	1 (2.3)	1 (10.0)	0 (0)	1 (6.3)	1 (6.3)	
	Black or Black other	1 (2.3)	0 (0)	0 (0)	0 (0)	0 (0)	
	Other / Unknown	7 (15.9)	3 (30.0)	0 (0)	2 (12.5)	0 (0)	
**BMI (kg/m^2)**		23.0 [21.7,28.8]	22.2 [21.0,25.8]	20.0 [17.8,24.0]	22.6 [20.7,25.8]	24.3 [20.5,26.8]	*0.339*
**Induction of labour (n)**	Yes	4 (9.0)	5 (50.0)	2 (33.3)	10 (62.5)	9 (56.3)	***<0.001 ****
	No	21 (47.7)	3 (30.0)	0 (0)	5 (31.3)	4 (25.0)	
	Unknown	19 (43.2)	2 (20.0)	4 (66.7)	1 (6.3)	3 (18.8)	
**Mode of delivery (n)**	SVD	23 (52.3)	5 (50.0)	1 (16.7)	10 (62.5)	7 (43.8)	*0.739*
	ELCS	11 (25.0)	2 (20.0)	2 (33.3)	2 (12.5)	3 (18.8)	
	EMCS	3 (6.8)	1 (10.0)	0 (0)	2 (12.5)	3 (18.8)	
	Instrumental	2 (4.5)	1 (10.0)	1 (16.7)	2 (12.5)	3 (18.8)	
	Unknown	5 (11.4)	1 (10.0)	2 (33.3)	0 (0)	0 (0)	
**Sex (n)**	Female	19 (43.2)	6 (60.0)	0 (0)	8 (50.0)	7 (43.8)	*0.292*
	Male	21 (47.7)	3 (30.0)	4 (66.7)	8 (50.0)	8 (50.0)	
	Unknown	4 (9.0)	1 (10.0)	2 (33.3)	0 (0)	1 (6.3)	
**Gestational age at delivery (weeks^{+days})**		39^{+4} [38^{+2},40^{+3}]	38^{+0} [37^{+2},39^{+2}]	36^{+2} [34^{+5},38^{+1}]	37^{+5} [36^{+5},38^{+4}]	37^{+5} [37^{+3},39^{+3}]	***<0.001 ****
**Birth weight (g)**		3345 [3014,3765]	2690 [2590,3235]	2760 [2573,2993]	3175 [2883,3438]	3255 [2970,3603]	***0.029 **
**Birth weight centile (%)**		63.0 [26.5,83.5]	34.0 [23.0,69.0]	71.0 [17.0,95.8]	84.0 [43.3,94.8]	71.0 [61.8,90.8]	*0.121*
**APGAR 1 minute ≤ 7 (n)**		2 (5.3)	0 (0)	0 (0)	0 (0)	0 (0)	*0.895*
**APGAR 5 minutes ≤ 7 (n)**		0 (0)	0 (0)	0 (0)	0 (0)	0 (0)	*n/a*
**Arterial pH ≤ 7.0 (n)**		0 (0)	0 (0)	0 (0)	0 (0)	0 (0)	*n/a*
**Venous pH ≤ 7.0 (n)**		0 (0)	0 (0)	0 (0)	0 (0)	0 (0)	*n/a*
**Non-reassuring CTG during labour (n)**		0 (0)	0 (0)	0 (0)	1 (6.3)	1 (6.3)	*0.107*
**Presence of meconium-stained amniotic fluid (n)**		3 (6.8)	1 (10)	0 (0)	1 (6.3)	1 (6.3)	*0.870*
**Admission to NNU (n)**		0 (0)	0 (0)	0 (0)	1 (6.3)	2 (12.5)	*0.590*

Table S3

			Control	Untreated Severe ICP	UDCA-treated Severe ICP	Untreated Mild ICP	UDCA-treated Mild ICP	*p*
**n**			**15**	**15**	**25**	**21**	**15**	
**Fetal NT-proBNP concentration (pg/L)**			1634 [981,4378]	6536 [1408,10500]	3511 [2243,7600]	3422 [1688,5186]	3108 [2374,4069]	***0.015 ****
**Fetal TSBA concentration at delivery (µmol/L)**			2.180 [1.75,2.85]	9.590 [5.20,18.12]	12.630 [3.45,26.50]	3.910 [3.19,7.31]	4.910 [2.01,7.39]	***<0.0001 *******
**Individual fetal serum bile acid concentrations at delivery (µmol/L)**	**CA**	unconjugated	0.03 [0.03,0.04]	0.06 [0.03,0.07]	0.06 [0.04,0.13]	0.05 [0.03,0.10]	0.05 [0.03,0.08]	***0.013 ****
		G-conjugated	0.25 [0.20,0.42]	1.84 [1.22,4.68]	1.31 [0.43,3.27]	0.83 [0.35,2.15]	0.36 [0.26,0.10]	***<0.0001 *******
		T-conjugated	0.39 [0.34,0.65]	2.03 [1.20,6.27]	1.13 [0.44,2.28]	0.97 [0.63,1.78]	0.70 [0.26,0.78]	***0.001 ******
	**CDCA**	unconjugated	0.02 [0.01,0.02]	0.02 [0.01,0.02]	0.02 [0.02,0.06]	0.02 [0.01,0.02]	0.02 [0.01,0.02]	***0.002 *****
		G-conjugated	0.51 [0.40,0.58]	1.72 [0.63,2.68]	2.05 [0.80,4.29]	0.72 [0.56,1.15]	0.98 [0.33,1.96]	***0.001 ******
		T-conjugated	0.80 [0.49,1.19]	1.41 [1.03,2.26]	0.87 [0.69,1.63]	1.01 [0.64,1.90]	0.76 [0.32,1.85]	*0.167*
	**DCA**	unconjugated	0.01 [0.00,0.01]	0.01 [0.00,0.02]	0.01 [0.00,0.02]	0.01 [0.00,0.04]	0.01 [0.00,0.01]	*0.835*
		G-conjugated	0.01 [0.01,0.02]	0.01 [0.01,0.04]	0.02 [0.01,0.03]	0.01 [0.01,0.02]	0.01 [0.01,0.02]	*0.677*
		T-conjugated	0.00 [0.00,0.01]	0.01 [0.00,0.04]	0.00 [0.00,0.02]	0.01 [0.00,0.02]	ND	*n/a*
	**LCA**	unconjugated	0.01 [0.00,0.01]	0.01 [0.00,0.01]	0.02 [0.01,0.02]	0.01 [0.00,0.02]	0.01 [0.01,0.02]	***0.001 ******
		G-conjugated	ND	ND	ND	ND	ND	*n/a*
		T-conjugated	ND	0.00 [0.00,0.01]	0.00 [0.00,0.01]	ND	0.00 [0.00,0.01]	*n/a*
	**UDCA**	unconjugated	0.00 [0.00,0.01]	0.00 [0.00,0.01]	0.87 [0.35,2.68]	0.00 [0.00,0.02]	0.20 [0.04,0.69]	***<0.0001 *******
		G-conjugated	ND	ND	2.33 [0.43,5.20]	0.00 [0.00,0.01]	0.36 [0.11,0.82]	*n/a*
		T-conjugated	ND	ND	0.24 [0.07,0.50]	ND	0.03 [0.02,0.09]	*n/a*
**Peak maternal TSBA concentration during pregnancy (µmol/L)**			3.0 [2.0,4.0]	71.0 [44.0,82.0]	94.0 [65.0,156.0]	22.0 [16.5,27.5]	31.0 [21.0,34.0]	***<0.0001 *******
**Peak maternal ALT concentration during pregnancy (IU/L)**			13.0 [10.0,21.0]	147.0 [52.0,361.0]	129.0 [95.0,256.0]	92.0 [34.5,201.0]	181.5 [33.0,290.5]	***<0.0001 *******
**Peak maternal bilirubin concentration during pregnancy (µmol/L)**			7.0 [5.0,8.0]	11.0 [7.0,12.0]	16.0 [13.0,23.0]	7.0 [5.0,8.5]	8.0 [6.0,12.5]	***<0.0001 *******
**Time between ICP onset and delivery (days)**			n/a	5.0 [3.0,24.0]	36.0 [27.5,58.5]	10.0 [5.5,30.0]	23.0 [11.0,33.0]	*n/a*
**UDCA dosage (mg)**			n/a	n/a	2000 [1250,2000]	n/a	1000 [1000,1500]	*n/a*
**Duration of UDCA treatment (days)**			n/a	n/a	36 [26,65]	n/a	12 [6,31]	*n/a*

Table S4

			Control	Untreated Severe ICP	UDCA-treated Severe ICP	Untreated Mild ICP	UDCA-treated Mild ICP	*p*
**n**			**43**	**10**	**6**	**16**	**16**	
**TSBA concentration at recording (µmol/L)**			1.0 [1.0,6.0]	53.5 [42.5,63.3]	78.5 [50.5,123.0]	19.5 [14.3,27.3]	21.5 [18.0,24.8]	***<0.001 ****
**ALT concentration at recording (IU/L)**			12.0 [11.0,15.0]	165.0 [95.8,350.0]	43.5 [10.8,116.0]	102.0 [75.3,200.3]	79.0 [33.3,120.0]	*0.072*
**Fetal cardiac time interval measurements (ms)**		**PR interval length**	117.0 [111.0,127.0]	133.0 [120.8,143.5]	128.5 [114.3,137.3]	119.5 [107.8,130.3]	79.0 [33.3,120.0]	***0.021 **
		**QTc interval length**	409.9 [384.2,426.3]	390.5 [352.2,425.0]	400.6 [357.2,418.5]	411.6 [372.6,428.2]	397.5 [357.0,422.1]	*0.680*
		**QRS Duration**	30.0 [26.0,34.0]	31.5 [28.8,34.0]	30.0 [28.8,33.0]	31.5 [30.0,33.0]	31.5 [30.0,33.8]	*0.758*
**Fetal heartrate variability measurements**	**Behavioural state 1F (quiet sleep)**	**RMSSD (ms)**	7.9 [6.7,9.6]	7.8 [6.7,8.9]	8.8 [7.1,10.4]	8.6 [6.9,10.5]	8.0 [7.0,8.7]	*0.763*
		**SDNN (ms)**	7.9 [5.0,9.7]	10.4 [9.2,17.5]	9.0 [5.8,12.2]	12.4 [9.5,14.5]	11.0 [7.3,13.5]	*0.062*
		**Median heartrate (bpm)**	129.9 [125.0,135.3]	128.1 [121.2,129.8]	121.9 [114.0,129.8]	126.9 [125.9,131.1]	128.5 [121.4,133.3]	*0.721*
	**Behavioural state 2F (active sleep)**	**RMSSD (ms)**	8.7 [7.6,9.6]	10.6 [8.8,11.4]	8.7 [7.1,11.0]	9.4 [8.8,11.1]	8.5 [7.6,9.7]	***0.049 **
		**SDNN (ms)**	18.2 [14.7,25.7]	26.2 [21.6,34.6]	19.1 [12.7,29.1]	25.4 [20.6,31.8]	21.9 [17.7,25.9]	***0.021 **
		**Median heartrate (bpm)**	134.5 [130.4,140.3]	130.5 [129.2,139.1]	132.1 [126.4,134.0]	133.2 [129.6,138.9]	133.4 [130.2,140.7]	*0.913*
**Maternal cardiac time interval measurements (ms)**		**PR interval length**	152.0 [139.0,170.5]	124.0 [117.5,152.5]	145.0 [140.5,154.8]	159.0 [143.3,166.8]	151.5 [140.8,166.5]	*0.087*
		**QTc interval length**	409.6 [396.1,433.3]	402.9 [357.1,415.8]	427.7 [373.3,452.7]	408.0 [382.6,415.6]	412.6 [388.8,425.2]	*0.357*
		**QRS Duration**	54.0 [49.5,59.5]	55.0 [49.5,70.0]	53.0 [48.0,62.0]	53.0 [46.8,56.5]	58.0 [52.5,61.8]	*0.320*
**Maternal heartrate variability measurements**		**RMSSD (ms)**	26.1 [16.6,37.2]	27.0 [19.9,38.7]	39.5 [29.6,53.9]	34.5 [24.2,42.0]	25.6 [19.5,43.1]	*0.208*
		**SDNN (ms)**	50.0 [41.7,59.2]	47.9 [34.9,61.6]	71.5 [43.7,77.9]	56.2 [36.3,66.7]	44. [27.6,59.9]	*0.189*
		**Median heartrate (bpm)**	74.9 [67.4,82.0]	73.2 [65.0,84.4]	72.3 [64.9,77.8]	72.7 [67.7,79.6]	69.1 [66.4,76.4]	*0.679*

Table S5

**Biochemical parameter in untreated ICP:**	**Positively correlated fetal cardiac parameter:**	**Brief description of cardiac parameter**
Peak maternal TSBA and ALT concentration during gestation	Fetal NT-proBNP concentration at delivery	NT-proBNP is a peptide hormone that is mainly secreted by the left ventricle of the heart and is commonly used as a biomarker of heart failure in adults. It is also associated with fetal distress, bradycardia and heart failure in fetuses.
Fetal bile acid hydrophobicity index at delivery	Fetal NT-proBNP concentration at delivery	
Fetal TSBA, GCA and TCA concentrations at delivery	Fetal NT-proBNP concentration at delivery	
Maternal TSBA concentration at time of ECG recording	Fetal PR interval length taken from ECG	The PR interval length on an ECG represents the time from atrial depolarisation to ventricular depolarisation during a single heartbeat. An elongated PR interval length is known to be associated with the incidence of atrioventricular block, a type of heart block resulting from impaired electrical conduction from the upper to the lower parts of the heart.
	Fetal RMSSD and SDNN values taken from ECG	RMSSD and SDNN are time-domain measures of heart rate variability. Changes in fetal heart rate variability are known to be associated with fetal autonomic nervous system dysfunction and fetal distress.

References

[1] Ghosh RE, Berild JD, Sterrantino AF, Toledano MB, Hansell AL. Birth weight trends in England and Wales (1986-2012): babies are getting heavier. Arch Dis Child Fetal Neonatal Ed 2018;103:F264-F270.

[2] Wacker-Gussmann A, Plankl C, Sewald M, Schneider KM, Oberhoffer R, Lobmaier SM. Fetal cardiac time intervals in healthy pregnancies - an observational study by fetal ECG (Monica Healthcare System). J Perinat Med 2018;46:587-592.

[3] Nijhuis JG, Prechtl HF, Martin CB, Bots RS. Are there behavioural states in the human fetus? Early Hum Dev 1982;6:177-195.

[4] Lucchini M, Wapner RJ, Chia-Ling NC, Torres C, Yang J, Williams IA, et al. Effects of maternal sleep position on fetal and maternal heart rate patterns using overnight home fetal ECG recordings. Int J Gynaecol Obstet 2020.
