# Supplementary material for: Fetal cardiac dysfunction in intrahepatic cholestasis of pregnancy is associated with elevated serum bile acid concentrations
Source: J Hepatol. 2021 May;74(5):1087–96. doi: 10.1016/j.jhep.2020.11.038 (PMC8062912; doi:10.1016/j.jhep.2020.11.038)
Supplement: CTAT table.pdf [file mmc2.pdf]

JOURNAL
OF HEPATOLOGY

Journal of Hepatology

CTAT methods

Tables for a "Complete, Transparent, Accurate and Timely account" (CTAT) are now mandatory for all revised submissions. The aim is to enhance the reproducibility of methods.

- Only include the parts relevant to your study
- Refer to the CTAT in the main text as 'Supplementary CTAT Table'
- Do not add subheadings
- Add as many rows as needed to include all information
- Only include one item per row

If the CTAT form is not relevant to your study, please outline the reasons why:

1.1 Antibodies

Name	Citation	Supplier	Cat no.	Clone no.

1.2 Cell lines

Name	Citation	Supplier	Cat no.	Passage no.	Authentication test method
N/A					

1.3 Organisms

Name	Citation	Supplier	Strain	Sex	Age	Overall n number
N/A						

1.4 Sequence based reagents

Name	Sequence	Supplier
N/A		

1.5 Biological samples

Description	Source	Identifier
91 umbilical venous serum samples	Queen Charlotte's Hospital and St. Thomas' Hospital	

1.6 Deposited data

Name of repository	Identifier	Link
N/A		

JOURNAL
OF HEPATOLOGY

1.7 Software

Software name	Manufacturer	Version
Monica DK	Monica Healthcare Limited (Nottingham, UK)	1.9
MATLAB	Mathworks Inc (US)	R2017a
Stata	Stata Corp (US)	15.0

1.8 Other (e.g. drugs, proteins, vectors etc.)

NT-proBNP (ELISA kit)	Supplier : RayBiotech, GA, USA	Cat no. : ELH-proBNP-1

1.9 Please provide the details of the corresponding methods author for the manuscript:

Professor Catherine Williamson
Maternal and Fetal Disease Group
Hodgkin Building, Guy's Campus
King's College London
SE1 1UL
020 7848 6014, catherine.williamson@kcl.ac.uk

2.0 Please confirm for randomised controlled trials all versions of the clinical protocol are included in the submission. These will be published online as supplementary information.

N/A
